# Complete mitochondrial genome sequencing and phylogenetic analysis of the Light-vented Bulbul (*Pycnonotus sinensis*) killed by window collision in South Korea in 2023

**DOI:** 10.1080/23802359.2025.2532040

**Published:** 2025-07-25

**Authors:** Dong-Yeop Lee, Heesu Lee, Ji-Yeon Hyeon, Dong-Hun Lee

**Affiliations:** a Wildlife Health Laboratory, College of Veterinary Medicine, Konkuk University, Seoul, Republic of Korea; b Infectious Diseases Laboratory, College of Veterinary Medicine, Konkuk University, Seoul, Republic of Korea

**Keywords:** Light-vented Bulbul, mitochondria, whole genome sequencing, phylogenetic analysis

## Abstract

The Light-vented Bulbul (*Pycnonotus sinensis*) has rapidly expanded across East Asia, particularly in South Korea since the early 2000s. This study presents the complete mitochondrial genomes of five individuals died from window collisions in Seoul. The genomes, sequenced using next-generation sequencing technology, are 16,923bp long and contain 13 protein-coding genes, 22 tRNAs, and 2 rRNAs. Phylogenetic analysis revealed that South Korean *P. sinensis* forms a monophyletic clade with *P. sinensis hainanus* from China, indicating high genetic similarity. These findings provide essential data for understanding of *P. sinensis* population structure and regional adaptation.

## Introduction

The Light-vented Bulbul (*Pycnonotus sinensis;* JF Gmelin, 1789), has rapidly expanded its range across East Asia in recent decades (Nakahara et al. 2024). On the Eurasian continent, its distribution has progressively extended northward since the 1930s, with a marked increase in the 1980s (Xing et al. 2013; Wen et al. 2014). In South Korea, the species was first recorded breeding on Socheong Island, South Korea, in 2004 (Moores 2007), and has continued to expand its range within the country (Nakahara et al. 2024). It has rarely been observed in Seoul, a metropolitan area in South Korea. This range expansion has been attributed to rising temperatures and the increased availability of human-made habitats (Wen et al. 2014). Despite the ongoing spread of *P. sinensis*, genetic information on this species remains limited. The mitochondrial genome has been reported as an effective tool for phylogenetic inference, phylogeography, and molecular evolution in birds (Kan et al. 2010). This study aims to determine the complete mitochondrial genome sequence of *P. sinensis* found in Seoul, South Korea, and to analyze its phylogenetic relationship with other *Pycnonotus* species worldwide.

## Materials and methods

**Figure 1.**
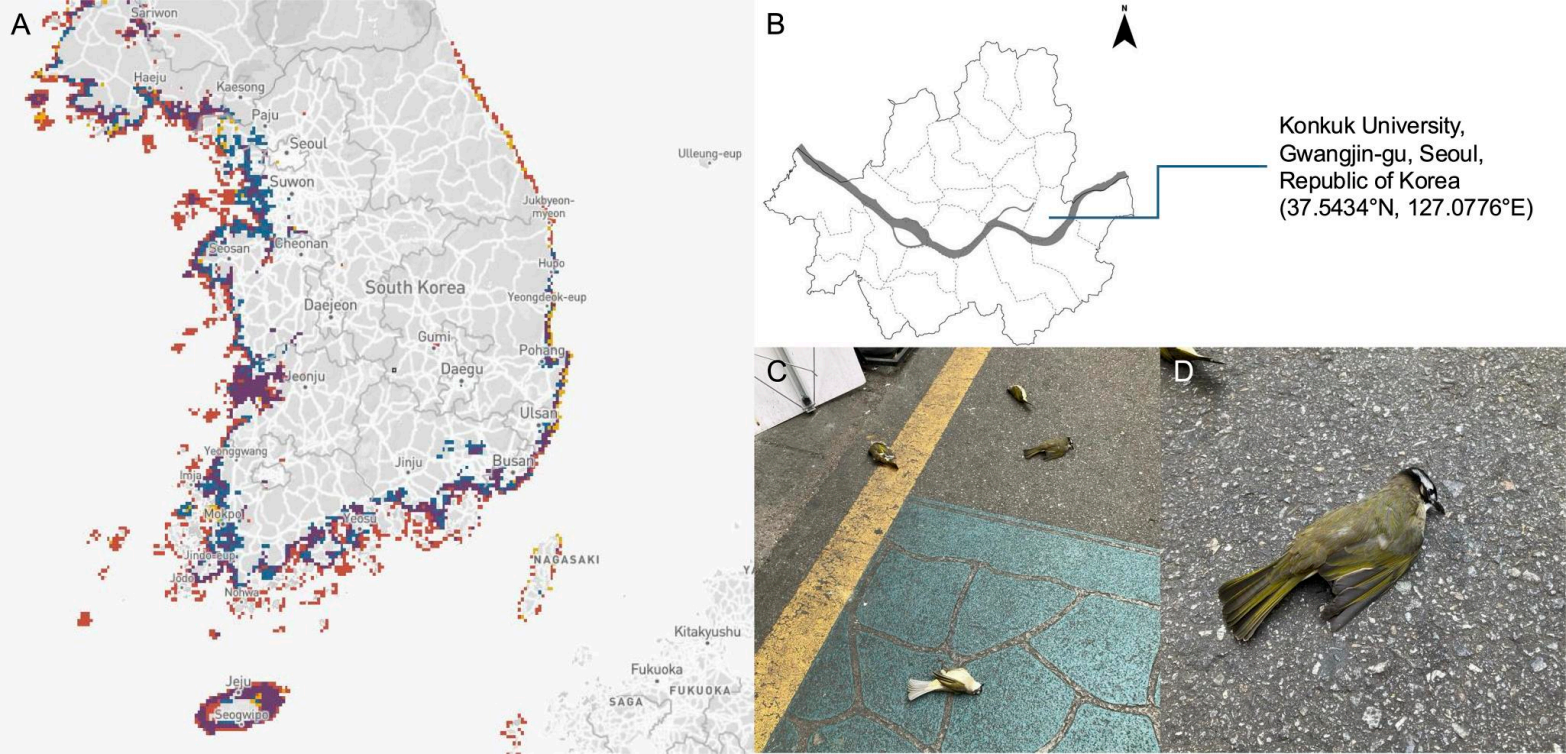
Geographic distribution, sampling location, and field photographs of *Pycnonotus sinensis* individuals collected for this study. (A) Distribution records of *P. sinensis* in South Korea based on eBird data (https://ebird.org), showing high relative abundance along coastal regions and Southern islands, as well as recent inland observations. Colors indicate seasonal status: purple – year-round presence, red – breeding season, blue – non-breeding season, yellow – migratory season. (B) Sampling location of window collision victims at Konkuk University, Gwangjin-gu, Seoul, Republic of Korea (37.5434°N, 127.0776°E). (C–D) Photographs of *P. sinensis* individuals found dead at the sampling site. Photographs were taken by Dong-Hun Lee.

In October 2023, five Light-vented Bulbuls, a species previously rarely observed in downtown Seoul, South Korea were found dead in the campus of Konkuk University (latitude 37.5434 N and longitude 127.0776E) (Figure 1B–D). The cause of death was determined to be window collisions, highlighting a growing concern regarding such incidents in urban environments. Initial species identification was based on external morphological features (e.g. white vent, black crown), and was subsequently confirmed through mitochondrial genome sequencing.

The birds underwent autopsy, and muscle tissue samples were collected for genomic DNA extraction using the DNeasy Blood & Tissue kit (Qiagen, Valencia, CA), following the manufacturer’s instructions. Specimens were deposited at College of Veterinary Medicine, Konkuk University (Prof. Dong-Hun Lee, E-mail: donghunlee@konkuk.ac.kr) under the voucher numbers bseq1-5.

The multiplex tiling PCR method was applied to amplify the genome of the samples using eleven pairs of tiling primers (Supplementary Table S1), which were designed based on a multiple alignment of previously published *P. sinensis* mitochondrial genomes to enable full-length genome coverage (Quick et al. 2017). Sequencing was performed using Illumina MiniSeq next-generation sequencing (NGS) system (Illumina, San Diego, CA).

Raw NGS reads were quality-filtered and trimmed using BBDuk v38.84 (https://sourceforge.net/projects/bbmap/), with a minimum quality threshold of 30. Both *de novo* and reference-based genome assembly were performed. For *de novo* assembly, trimmed reads were assembled using the SPAdes assembler version 3.15.5 (Prjibelski et al. 2020). For reference-based assembly, trimmed reads were mapped to the *P. sinensis* mitochondrial genome (GenBank accession number: NC_013838) using Minimap version 2.24 with default settings, and the results were visualized using Geneious Prime (Li 2018). Final consensus sequences were obtained by integrating results from both assembly approaches. Following the previously described protocol (dx.doi.org/10.17504/protocols.io.4r3l27jkxg1y/v1), we assessed the assembly’s accuracy by analyzing the sequencing coverage depth (Supplementary Figure S1). Finally, the complete sequence was annotated using the MITOS2 web server (Bernt et al. 2013).

For phylogenetic analysis, complete mitochondrial genome sequences (*n* = 10) of Pycnonotidae species and one *Pterorhinus chinensis* (outgroup) were downloaded from GenBank. Sequences were aligned using MAFFT v7.490 (Katoh and Standley 2013), and a maximum likelihood (ML) phylogeny was constructed using IQ-TREE 2 with automatic model selection (Minh et al. 2020). Node support was assessed using 1,000 ultrafast bootstrap replicates. The resulting phylogeny was visualized using Interactive Tree of Life (v7.2) (Letunic and Bork 2024).

## Results

**Figure 2.**
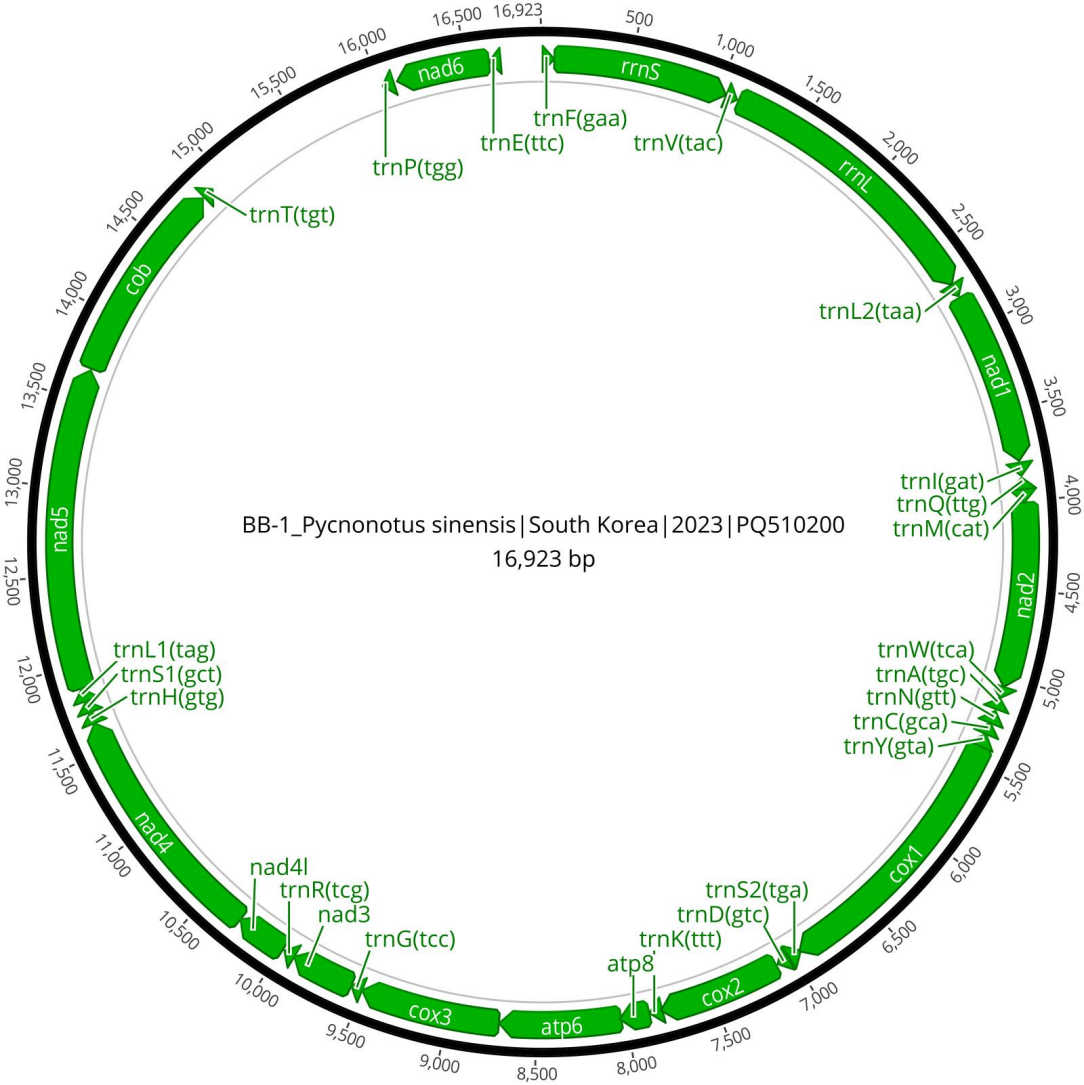
Mitochondrial genome map of *Pycnonotus sinensis* (GenBank accession number: PQ510200).

The complete mitochondrial genome sequences of *P. sinensis* obtained in this study were 16,923bp in length and encoded 13 protein-coding genes, 22 transfer RNA (tRNA) genes, and 2 ribosomal RNA (rRNA) genes (Figure 2). The nucleotide composition was as follows: *A* = 30.6%, *T* = 23.3%, *G* = 14.4% and *C* = 31.7%. Sequence identity among the five individuals ranged from 99.65% to 99.77%, indicating a close genetic relationship within this local population.

**Figure 3.**
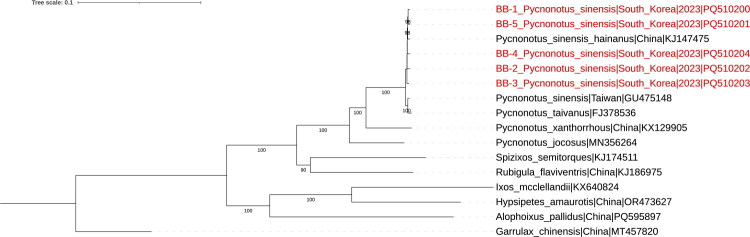
Maximum likelihood phylogeny of complete *Pycnonotus sinensis* mitochondrial genome. The percentages of the bootstrap test in 1,000 replicates were shown above the branches. The following sequences were used: GenBank accession: PQ510200–PQ510204 (this study), KJ147475 (Ren et al. 2016), KJ174511 (Ren et al. 2016), KJ186975 (Ren et al. 2016; Jha et al. 2021), KX129905 (Wen and Liao 2016), OR473627 (Li et al. 2024), MN356264 (Feng et al. 2020), GU475148 (GenBank direct submission), FJ378536 (GenBank direct submission), KX640824 (GenBank direct submission), and PQ595897 (GenBank direct submission). *Pterorhinus chinensis* (accession no. MT457820) (Bai et al. 2023) mt-genome was used as an outgroup in the phylogenetic analysis. The mt-genome sequenced in this study are indicated with red taxa. The scale bar indicates nucleotide substitutions per site.

Phylogenetic analysis revealed that the *P. sinensis* sequences from this study formed a monophyletic group with *P. sinensis hainanus* from China (GenBank accession number: KJ147475), supported by a high bootstrap value (98%) and high nucleotide sequence identity (99.62–99.69%) (Figure 3). This clade was distinct from the mitogenome sequences of *P. taivanus* (FJ378536) and *P. sinensis* from Taiwan (GU475148). The five South Korean mitogenomes diverged by 0.73–0.79% from *P. taivanus* (FJ378536) and by 0.54–0.60% from Taiwanese *P. sinensis* (GU475148).

These findings are consistent with the haplotype network analysis conducted by McKay et al. (2013), which identified two major haplotype clusters: one consisting of *P. s. sinensis* and *P. s. hainanus,* and the other consisting of *P. s. formosae*, *P. s. orii* and *P. taivanus* (McKay et al. 2013).

## Discussion and conclusion

Previously a complete mitogenome of *Pycnonotus sinensis hainanus* identified in China was reported (Ren et al. 2016), but genetic information of this species remains limited. The present study contributes novel complete mitogenomes (*n* = 5) of individuals sampled in Seoul, South Korea, which improve phylogeographic resolution of this species. These specimens, collected from window collision mortality events in a metropolitan city, provide valuable insights into both the ongoing range expansion of *P. sinensis* and the conservation challenges posed by urban environments.

It has been suggested that the migration of the *Pycnonotus sinensis* to the Korean Peninsula is likely driven by environmental changes in China, with the species relocating due to climate fluctuations in their native habitats (Park and Choi 2018). Since its initial observation in South Korea in 2002, the number of recorded sightings of *P. sinensis* has increased significantly (Nakahara et al. 2024). First documented on Eocheong Island in October 2002, subsequent sightings and breeding records during the 2000s were predominantly confined to the remote islands of the Yellow Sea (Park and Choi 2018). Gradually, reports extended to coastal regions of the Korean Peninsula. By the late 2010s, flocks of *P. sinensis* were observed not only on remote islands but also in major urban areas such as Seoul and Busan (Nakahara et al. 2024).

A recent study suggested that the northward range expansion of Chinese bulbuls across the Eurasian continent is associated with rising temperatures and the increasing availability of human-modified habitats (Wen et al. 2014). In South Korea, the mean annual temperature has increased by 1.7 °C over the past century, including a 1.2 °C increase from 1997 to 2014 alone (Park and Choi 2018). This warming may act as an ecological driver, enabling subtropical species to colonize previously unsuitable northern habitats (Wen et al. 2014).

Distribution records of *P. sinensis* in South Korea based on eBird data (https://ebird.org) show high relative abundance along coastal regions and southern islands, as well as recent inland observations (Figure 1A). Notably, the individuals analyzed in this study were collected in metropolitan Seoul and are presumed to have died from window collisions. *P. sinensis* is considered a habitat generalist and urban adapter among Passeriformes in East Asia (Wang et al. 2009). Chinese bulbuls exhibit behavioral flexibility that supports their adaptation to urban environments. This includes the use of artificial food resources, modification of nesting materials, and development of diverse microgeographic song dialects (Chen et al. 2022). Recent studies have shown that nest predation pressure tends to decrease with the level of urbanization, due to reduced natural predator presence and increased availability of complementary food sources, which improve physical condition and reduce energetic demands on parents (Chen et al. 2022). Furthermore, feeding frequency in Chinese bulbuls has been shown to increase with urbanization indices, suggesting that urban areas provide more abundant resources for rearing offspring (Chen et al. 2023).

Cities with extensive anthropogenic landscapes and numerous man-made structures have experienced a notable increase in bird-window collisions, now recognized as one of the leading causes of avian mortalities worldwide (Basilio et al. 2020). The Korean National Institute of Ecology estimates that approximately 7.88 million birds die annually in South Korea due to collisions, with 7.65 million of these deaths resulting from collisions with building windows and an additional 0.23 million from collisions with transparent noise barriers (Kim et al. 2023). The presence of tall buildings, glass windows, and noise barriers may significantly impact the wild bird populations and their ecology. Future conservation efforts could involve implementing bird-friendly architectural designs, such as patterned or UV-reflective glass, and public education programs to raise awareness of urban bird collisions.

While mitochondrial DNA provides useful information for species identification and phylogenetic inference, it does not capture genome-wide variation. The present study focused on mitogenome-based phylogenetic placement, and did not incorporate nuclear markers or genome-wide data, which limits our ability to assess population structure or adaptive divergence in detail.

In summary, we sequenced the complete mitochondrial genome of *P. sinensis* and conducted a phylogenetic analysis alongside other species of *Pycnonotus*. This study provides valuable genetic data for a comprehensive understanding of *P. sinensis* populations that have expanded their habitat in South Korea. Future research should include broader geographic sampling, long-term monitoring of urban populations, and genomic studies incorporating nuclear and functional markers to assess population differentiation, adaptation, and the ecological consequences of urban colonization.

## Supplementary Material

supplementary_table_1.docx

supplementary_figure.docx

## Data Availability

The genome sequence data that support the findings of this study are available in GenBank of NCBI at https://www.ncbi.nlm.nih.gov/ under the accession no. PQ510200-PQ510204. The NGS library and tissue samples are stored at the College of Veterinary Medicine, Konkuk University. The associated BioProject, SRA numbers are PRJNA1220190, and SRR32261902-SRR32261906. The associated Bio-Samples numbers are SAMN46714156, SAMN46714321, SAMN46714322, SAMN46714430, and SAMN46714432.
