# Supplementary material for: Complete mitochondrial genome sequencing and phylogenetic analysis of the Light-vented Bulbul (Pycnonotus sinensis) killed by window collision in South Korea in 2023
Source: Mitochondrial DNA B Resour. 2025 Jul 25;10(8):773–7. doi: 10.1080/23802359.2025.2532040 (PMC12302383; doi:10.1080/23802359.2025.2532040)
Supplement: supplementary_table_1.docx [file TMDN_A_2532040_SM0675.docx]

**Table S1. List of primers used to amplify and sequence the mitochondrial genome of *Pycnonotus sinensis***

| Fragment name | Primer name | Direction^a^ | Sequence (5’-3’) | Nucleotide position^b^ |
| --- | --- | --- | --- | --- |
| 1 | bulbul_MT_1_LEFT | F | TGTCAAGATGGCTGCTACATGC | 37-2,035 |
|  | bulbul_MT_1_RIGHT | R | CGCGGCCGTTAAACATTATGTC |  |
| 2 | bulbul_MT_2_LEFT | F | GACGCAAGCTTACATCCGTACA | 1,797-3,801 |
|  | bulbul_MT_2_RIGHT | R | AGGCACATTTCCTTGGTCATCC |  |
| 3 | bulbul_MT_3_LEFT | F | AACCCTTCTCCTAACAGCAGGA | 3,610-5,606 |
|  | bulbul_MT_3_RIGHT | R | TATTACGAAGGCATGGGCTGTG |  |
| 4 | bulbul_MT_4_LEFT | F | CCACAAAGACATCGGCACCTTA | 5,444-7,397 |
|  | bulbul_MT_4_RIGHT | R | TTTAGGGTCAGGTCTGGTTCGT |  |
| 5 | bulbul_MT_5_LEFT | F | ATTCCACGACCACGCTCTAATG | 7,173-9,215 |
|  | bulbul_MT_5_RIGHT | R | AGAACTGTTAGGGTAAGGGCGT |  |
| 6 | bulbul_MT_6_LEFT | F | AGCCTTCTTCCACTCAAGCCTA | 9,009-11,030 |
|  | bulbul_MT_6_RIGHT | R | TTTGCTAGGAGGGTGAGTCGTA |  |
| 7 | bulbul_MT_7_LEFT | F | ACTACCTCTTGATCCAGCCTCA | 10,837-12,849 |
|  | bulbul_MT_7_RIGHT | R | ATGCAGGAATGCTAGTTGTGGG |  |
| 8 | bulbul_MT_8_LEFT | F | AACTGCCCTAACCCTATGCCTA | 12,678-14,659 |
|  | bulbul_MT_8_RIGHT | R | ATTTGTGATAGGGGGCGGAATG |  |
| 9 | bulbul_MT_9_LEFT | F | CCCCCACACATTAAACCCGAAT | 14,484-16,443 |
|  | bulbul_MT_9_RIGHT | R | GGGGTATGTTGGTGGTGTTTGT |  |
| 10 | bulbul_MT_10_LEFT | F | CCCCTTCTTAGAGTTCGCAACAA | 14,889-16,845 |
|  | bulbul_MT_10_RIGHT | R | TGGTGTGTGGATGGTAGTCTGT |  |
| 16367-58 | bulbul_16367_F | F | ACCTCAATCAGCTCAAGCCT | 16,367-58 |
|  | bulbul_58_R | R | GCATGTAGCAGCCATCTTGAC |  |

^a^ F and R, forward and reverse direction of transcription

^b^ Nucleotide positions are with respect to *Pycnonotus sinensis* mitochondrial genome.
