# Supplementary material for: Complete mitochondrial genome sequencing and phylogenetic analysis of the Light-vented Bulbul (Pycnonotus sinensis) killed by window collision in South Korea in 2023
Source: Mitochondrial DNA B Resour. 2025 Jul 25;10(8):773–7. doi: 10.1080/23802359.2025.2532040 (PMC12302383; doi:10.1080/23802359.2025.2532040)
Supplement: supplementary_figure.docx [file TMDN_A_2532040_SM0674.docx]

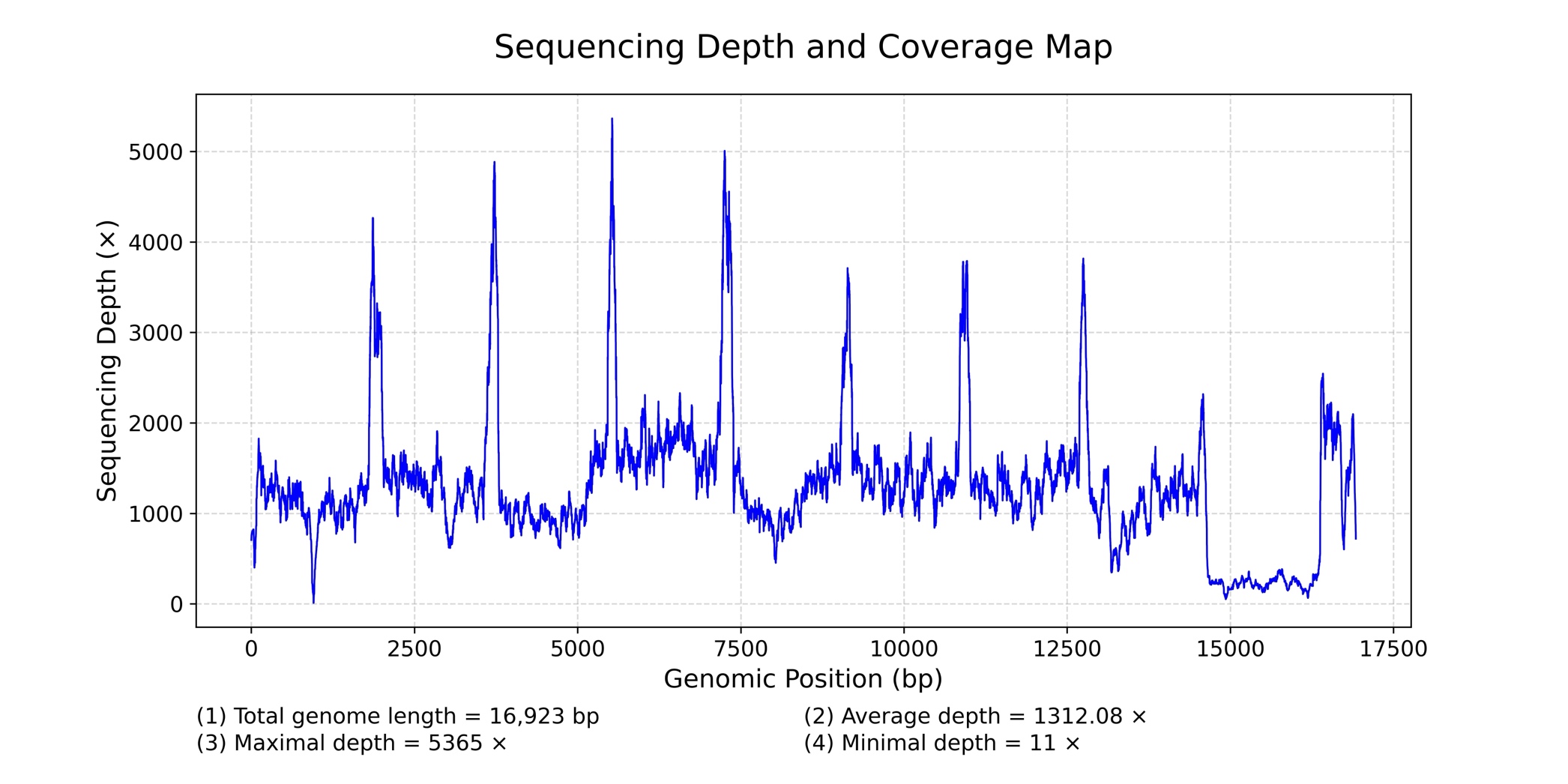


Figure S1. The estimated coverage depths from Illumina sequencing reads for *Pycnonotus sinensis* mitogenome (GenBank accession number: PQ510200)


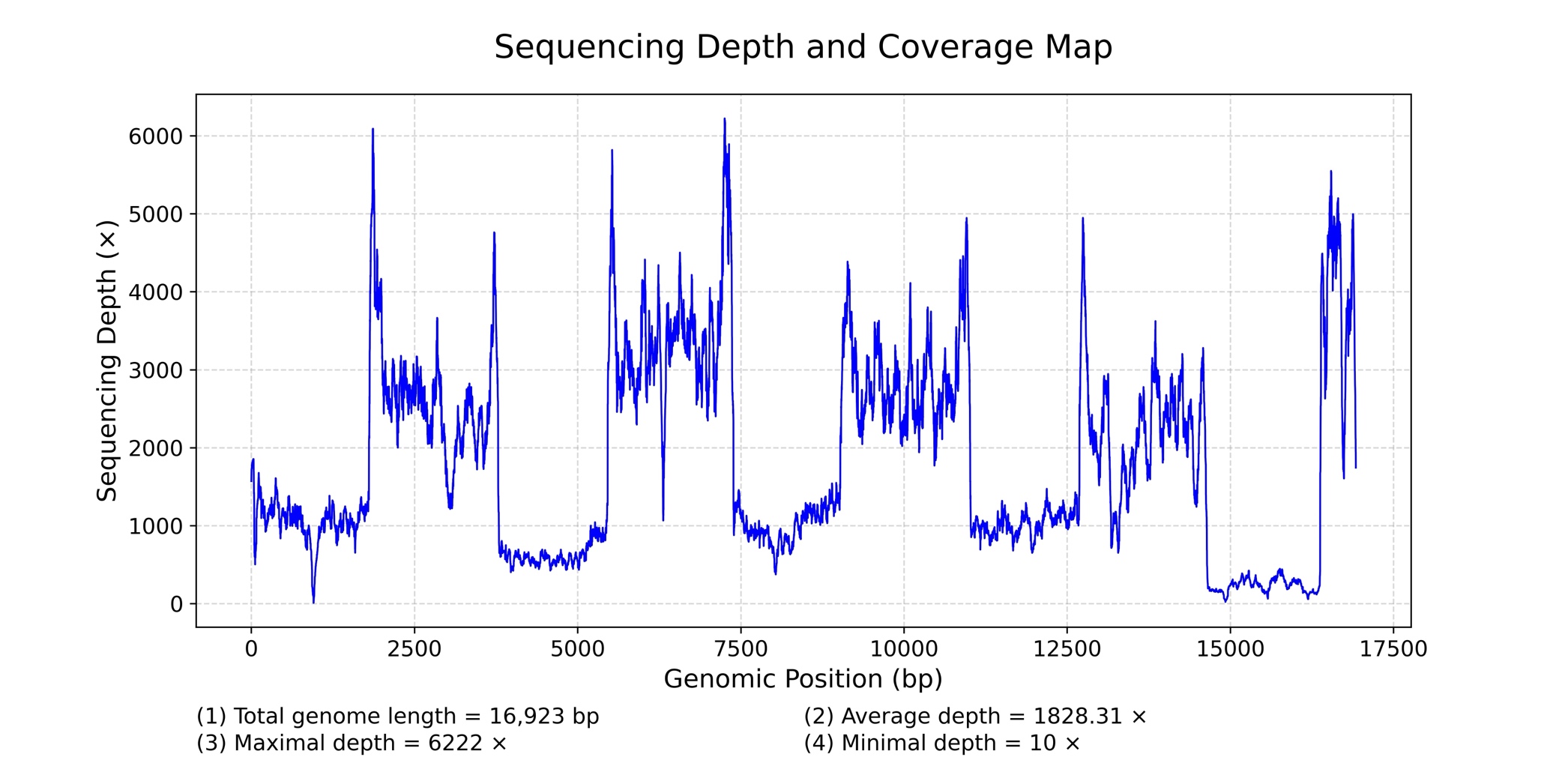


Figure S2. The estimated coverage depths from Illumina sequencing reads for *Pycnonotus sinensis* mitogenome (GenBank accession number: PQ510201)


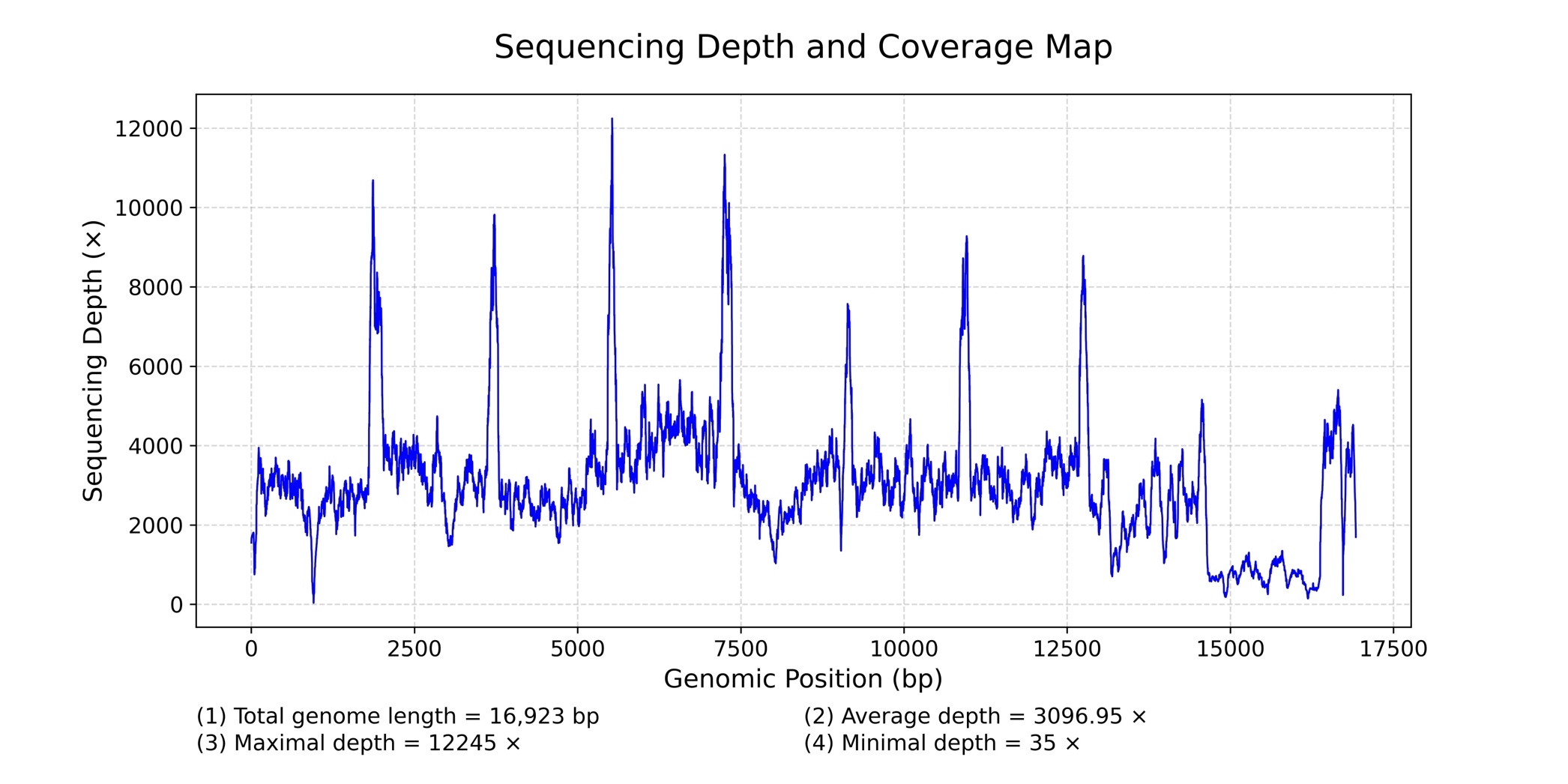


Figure S3. The estimated coverage depths from Illumina sequencing reads for *Pycnonotus sinensis* mitogenome (GenBank accession number: PQ510202)


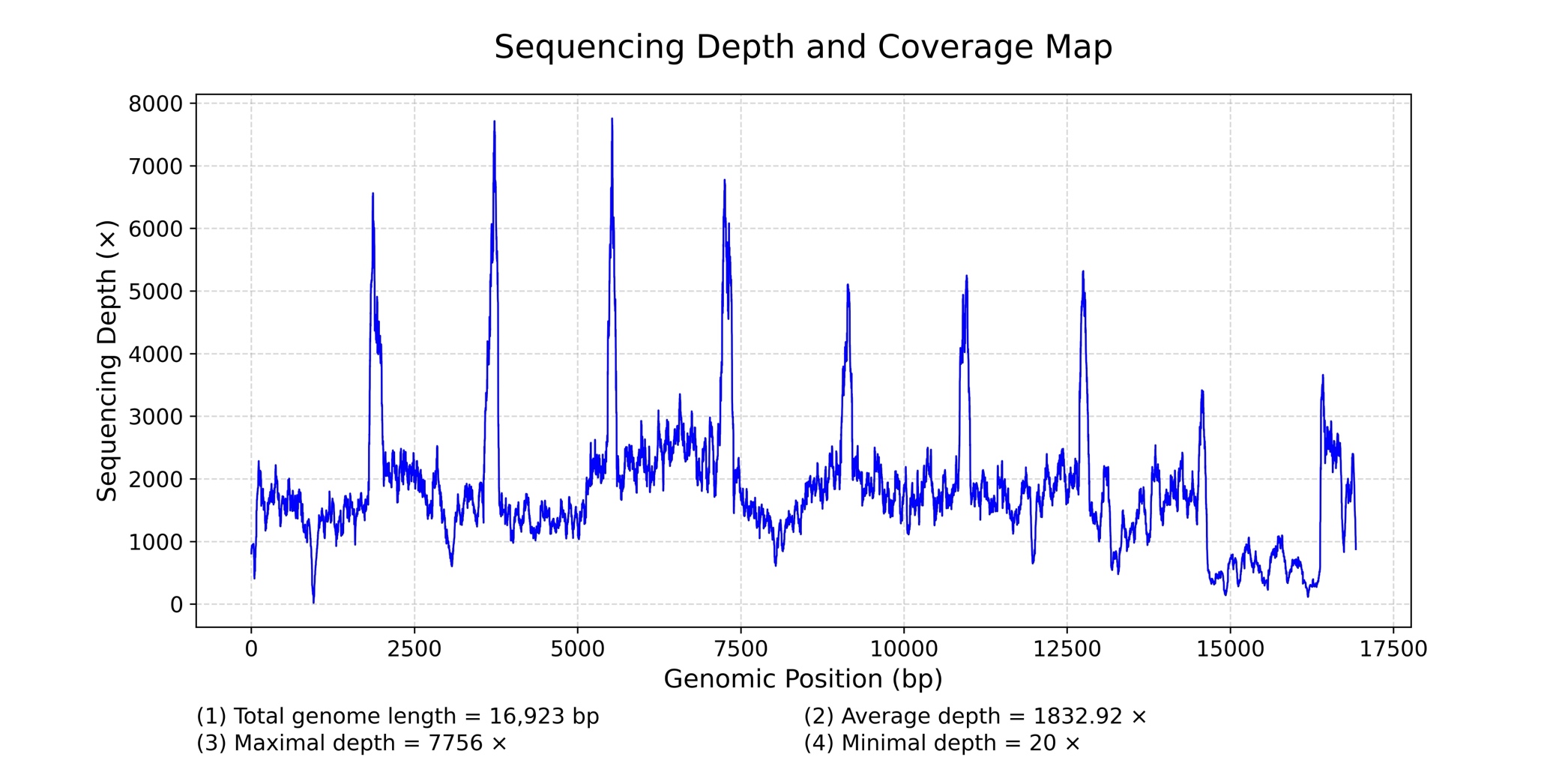
 Figure S4. The estimated coverage depths from Illumina sequencing reads for *Pycnonotus sinensis* mitogenome (GenBank accession number: PQ510203)


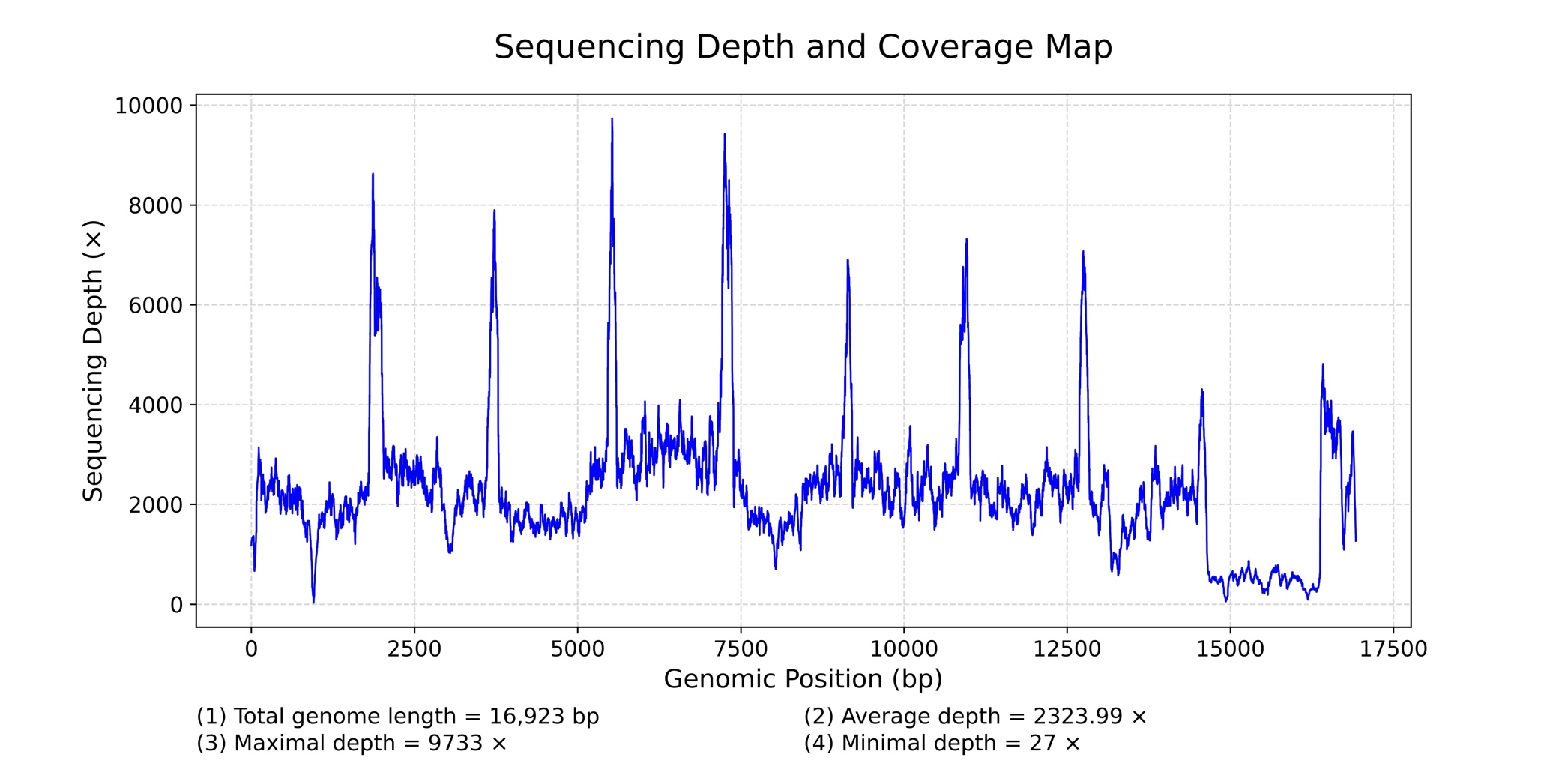
 Figure S5. The estimated coverage depths from Illumina sequencing reads for *Pycnonotus sinensis* mitogenome (GenBank accession number: PQ510204)
